# A Novel Variant of Adenosine Deaminase 2 Deficiency Presented With Chronic Thrombocytopenia, Anemia, and Early-Onset Stroke

**DOI:** 10.7759/cureus.15288

**Published:** 2021-05-28

**Authors:** Abdulqader Al-Hebshi, Maher Aljohani, Naif AlShenaifi, Maryam Aloqbi, Waheed Turkistani, Fahad Hakami

**Affiliations:** 1 Pediatric Hematology Oncology, Ministry of National Guard Health Affairs, Medina, SAU; 2 Pediatrics, King Saud Bin Abdulaziz University for Health Sciences, Riyadh, SAU; 3 Pathology, Taibah University, Medina, SAU; 4 Pediatrics, Prince Mohammed Bin Abdulaziz Hospital, Ministry of National Guard Health Affairs, Medina, SAU; 5 Pathology and Laboratory Medicine, Prince Mohammed Bin Abdulaziz Hospital, Ministry of National Guard Health Affairs, Medina, SAU; 6 Pediatrics, King Salman Medical City, Ministry of Health, Medina, SAU; 7 Pathology and Laboratory Medicine/Genetics, King Abdulaziz Medical City, Jeddah, SAU

**Keywords:** stroke, ada2, vasculitis, anemia, thrombocytopenia

## Abstract

Deficiency of adenosine deaminase 2 (DADA2) is a rare recessive disorder caused by the bi-allelic loss-of-function pathogenic variants in the *ADA2* gene (MIM: 607575, also known as *CECR1*, cat eye syndrome chromosome region, candidate 1). Based on the Human Gene Mutation Database (HGMD^®^), 53 different disease-causing variants have been identified in this gene to date. This case report aims to describe a new vasculitis, autoinflammation, immunodeficiency, and hematologic defects syndrome (VAIHS) case caused by a novel pathogenic variant. A four-year-old boy was referred to our hospital with anemia, thrombocytopenia, and stroke, but no skin manifestations. The patient had a significant phenotypic overlap with VAIHS. Molecular genetic analysis via whole exome sequencing identified a homozygous deleterious variant in *ADA2*. To our knowledge, the identified variant has never been described in the literature. Screening for *ADA2* pathogenic variants should be considered in the differential diagnosis of pediatric patients manifesting with chronic thrombocytopenia or early-onset stroke for an accurate diagnosis and appropriate treatment choices.

## Introduction

Deficiency of adenosine deaminase 2 (DADA2) is a rare autosomal-recessive disorder caused by bi-allelic loss-of-function pathogenic variants in the *ADA2 *gene (MIM: 607575, also known as CECR1, cat eye syndrome chromosome region, candidate 1). This disease was firstly described in 2014 and is now known as vasculitis, autoinflammation, immunodeficiency, and hematologic defects syndrome (VAIHS). The clinical findings reported were systemic vasculitis with fever, livedo racemosa, and early-onset lacunar strokes mimicking polyarteritisnodosa (PAN) [1,2]. The clinical spectrum of this condition has expanded considerably. Hematological features, such as pure red cell aplasia (PRCA), thrombocytopenia, lymphopenia, neutropenia, and aplastic anemia have been reported in several DADA2 patients [3]. Less common manifestations were abnormalities in the arteries of the gastrointestinal system, hypogammaglobulinemia, and vision problems [4]. Diseases associated with *ADA2 *include Sneddon syndrome and VAIHS. Broad multidisciplinary studies clarified the pathogenesis and highlighted the advancement of enzyme replacement and gene treatment [5,6].

Based on the Human Gene Mutation Database (HGMD®), 53 different disease-causing variants have been identified in this gene. The clinical course and severity of the clinical presentation range considerably, even in patients of the same family [7].

The following case report describes a new VAIHS case, caused by a novel pathogenic variant.

## Case presentation

A four-year-old boy was referred to our hospital with chronic immune thrombocytopenia, hemolytic anemia, and positive direct antiglobulin test (Table 1), and labeled as Evans syndrome. His medical history indicated that the patient had an unremarkable history until the age of 18 months, when he presented with multiple skin bruises and spontaneous gum bleeding, along with pale appearance and fatigue. There was no history of weight loss, recurrent infections, or body ache/bone pain. He is a product of consanguineous marriage, has one sister, and none of his family members had any significant medical history or any blood disorder.

**Table 1:** Complete blood count and direct agglutinin test results WBC: white blood cell; DAT: direct agglutinin test; Hb: hemoglobin

WBC	3.7x 10^9^/L (normal 5-12 × 10^9^ cells/L)
Hb (g/L)	5.7 g/dL (normal 12 to 16 g/dL)
Platelet	3 x10^9 ^/L (normal 150 – 450 × 10^9^ cells/L)
Reticulocyte count	15% (normal 0.5-1.5%)
DAT	Positive

Upon the initial diagnosis at age of 18 months, the patient was treated with two doses of intravenous immunoglobulin (IVIG) 1 mg/kg/dose and a high dose of intravenous methylprednisolone, 30 mg/kg once daily for three days. The platelet count increased to 50x10^9^/L, but the hemoglobin dropped to 5 g/dl, mandating a blood transfusion. After a week, the patient was discharged home with a platelet count of 316x10^9^/L and a hemoglobin (Hb) level of 9.5 g/dl. He was discharged on oral prednisolone, 2 mg/kg/day for one week, which was tapered over 21 days. During the tapering period, he developed an ecchymotic skin rash and mild epistaxis. The platelet count dropped to 30x10^9^/L, and he was kept on 5 mg oral prednisolone every other day for one year to control his symptoms.

**Figure 1:**
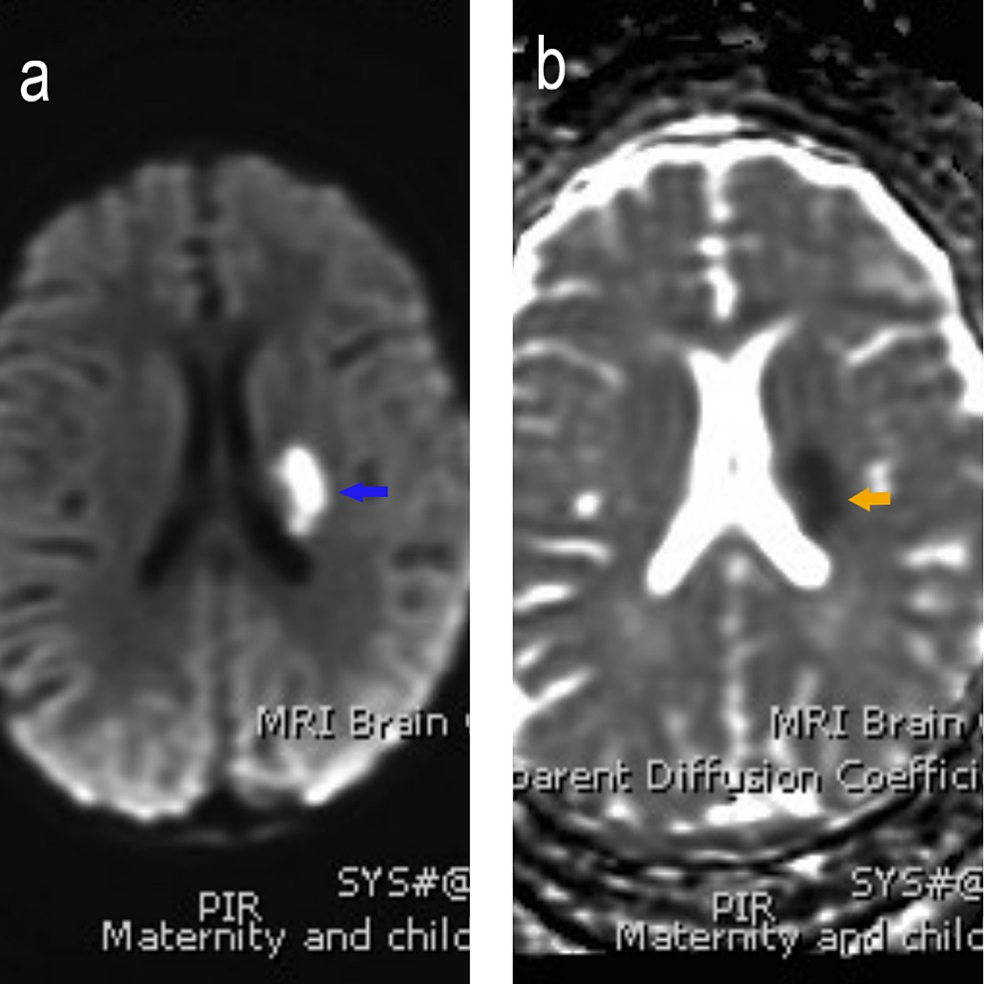
The MRI brain shows (a) bright signal (blue arrow) of the left basal ganglia on diffusion-weighted imaging (DWI), and (b) black signal (orange arrow) on the apparent diffusion coefficient map (ADC map), showing restricted diffusion, which indicates acute infarction.

At the age of two years, the patient developed a seizure, followed by slurred speech and right-sided weakness for two weeks. His platelet count was 270x10^9^/L (normal 150-450x10^9^/L), and the Hb was 11 g/dl (normal 11.0-14.5 g/L). Brain magnetic resonance imaging (MRI) showed evidence of an altered signal at the left basal ganglia with an internal capsule, indicating an acute/subacute basal ganglia infarction (Figure 1). He received intravenous unfractionated heparin for two days, followed by oral aspirin 81 mg daily for 7 months.

**Figure 2:**
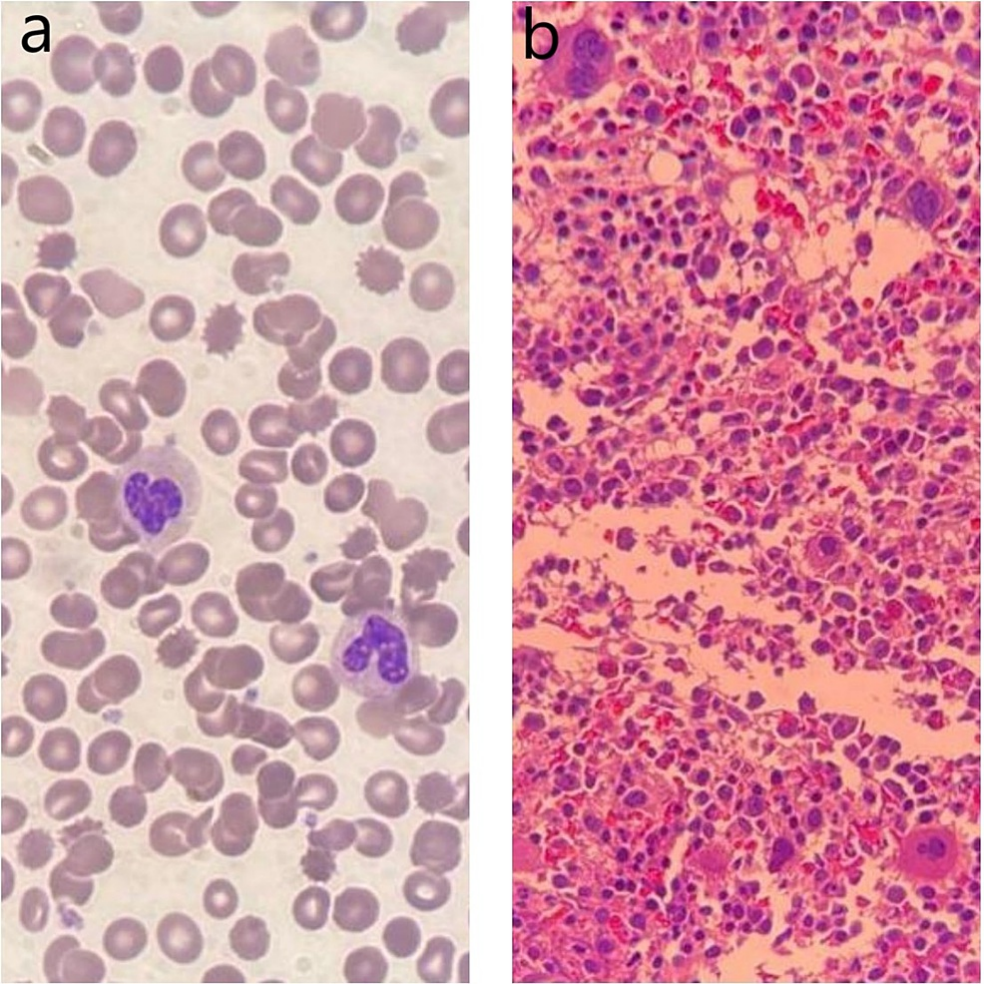
(a) The patient’s laboratory results via a Wright-Giemsa stained peripheral blood smear revealed leukopenia and severe neutropenia, with a normal RBC and platelet count. The corrected reticulocyte count was reduced. (b) A bone marrow trephine biopsy section demonstrated a mildly hypocellular marrow tissue for the patient’s age, heterocellular distribution of hematopoiesis, and no abnormal cell collections or fibrosis. RBC: red blood cell

**Figure 3:**
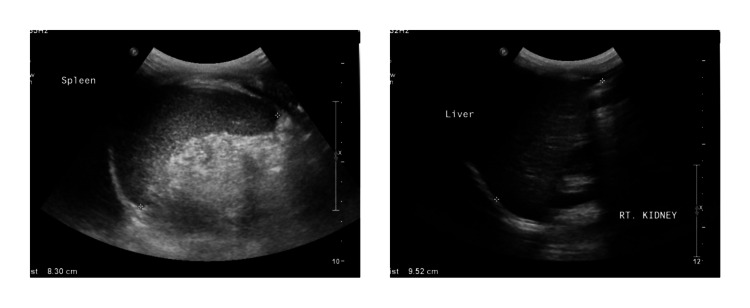
Ultrasound scan shows mild enlargement of the liver (right) and spleen (left).

The patient presented at our hospital with a history of fever and upper respiratory tract infection. The patient had an initial heart rate of 118 beats/min with normal blood pressure. His respiratory and cardiac examination was normal, but he had hepatosplenomegaly (spleen and liver were palpable 3 cm below costal margins) and multiple ecchymoses of the upper and lower limbs. The central nervous system examination was normal, and no other system was affected. Laboratory tests reported thrombocytopenia, anemia, neutropenia, and reticulocytopenia. The platelets were 2x10^9^/L, and the Hb 9 g/dl, absolute neutrophil count (ANC) 0.4x10^9^/L (normal 0.80-5.40x10^9^/L), reticulocyte count 0.2% (normal 0.5-1.5%), no evidence of hemolysis, and a negative direct agglutinin test (DAT). The patient's blood smear showed leukopenia, severe neutropenia, some atypical white blood cells, and moderate-to-severe microcytic anemia. However, the flow cytometry analysis of the bone marrow reported no immunophenotypic evidence of an abnormal phenotype. His bone marrow evaluation revealed mild hypocellularity (60-70% cellular) for the patient’s age. There were no abnormal cell collection or apparent fibrotic megaloblastic/megaloblastoid changes (4-5%) and no morphological evidence of acute leukemia (Figure 2). The serum folate, vitamin B12 and serum complement levels were normal. The viral and bacterial serology, including rubella virus, hepatitis b virus, cytomegalovirus, Epstein-Barr virus, lupus, and the tuberculin skin test were all negative. There was hypogammaglobulinemia, immunoglobulin M (IgM) 0.12 g/L (normal 0.40-1.50), low levels of IgA 0.12 g/L (normal 0.30-1.50) and a low IgG level 3.84 g/L (normal 3.40-13.60). Imaging studies included a chest X-ray, which was normal. The abdominal ultrasound revealed a mildly enlarged spleen (8.30 cm) and liver (9.52 cm), a normal outline surface, and no focal lesions were seen (Figure 3).

**Figure 4:**
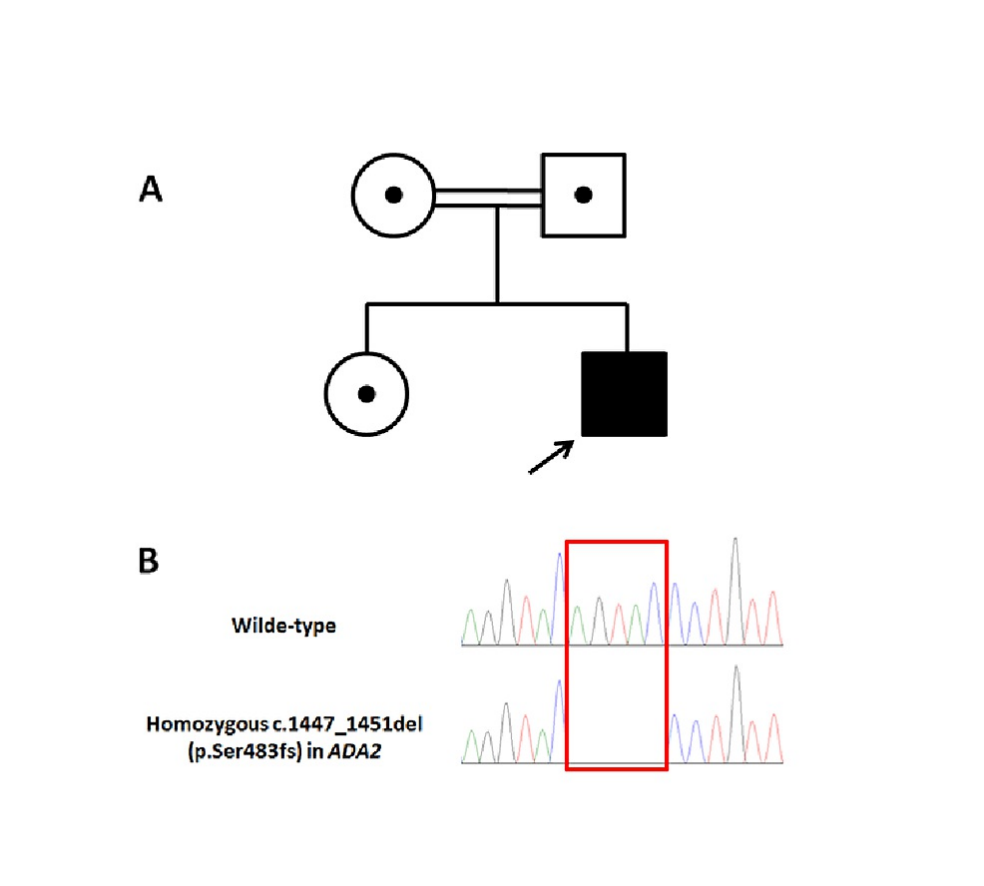
A) Pedigree diagram, showing consanguineous parents with one affected and one unaffected child. B) Sanger confirmation showing the homozygous deletion in the affected child.

A clinical diagnosis of VAIHS was suggested. However, to confirm the diagnosis and for appropriate family counseling, genetic analysis via whole-exome sequencing (WES) was requested and performed in a reference laboratory. The WES confirmed the diagnosis and identified a novel homozygous 5 base pair deletion in *ADA2 *[NM_001282225.2: c.1447_1451del (p.Ser483fs)]. It had no record in literature or any of the general population studies, including the Genome Aggregation Database (gnomAD) and the Exome Aggregation Consortium (ExAC). This variant has been added by our institution to the ClinVar database (Accession: VCV000800755.1). Sanger segregation analysis showed that both parents and the unaffected sister are heterozygous for this variant (Figure 4).

The patient received packed red blood cells and IVIG, 1 g/kg for two days. He was prescribed oral eltrombopag (a thrombopoietin receptor agonist) 50 mg once daily and granulocyte colony-stimulating factor (G-CSF) at a dose of 50 mic/kg as a subcutaneous injection. He responded well after three days. Upon discharge, he was clinically stable, his Hb level was 11 g/dl, platelet count was 50x10^9^/L, and ANC was 1.47x109/L. As the patient did not have alarming manifestations, serial follow-up with continued steroid therapy was considered. The patient was transferred to a higher center to follow pediatric rheumatology and bone marrow transplant team for further evaluation and management.

## Discussion

To our knowledge, this variant and the allele frequency in the general population has not yet been described in the literature. It was also the first detection in our internal database. Considering the available information, the variant is classified as likely pathogenic.

Despite the absence of confirmation by a larger investigation, preliminary studies reported that undetectable *ADA2* activity was associated with a more severe clinical course [8]. The predisposition of heterozygosity of the *ADA2 *mutation to late-onset cardiovascular diseases must still be established. There is no relationship between common gene variants and vascular disorders through genome-wide association studies (GWAS) [9].

Due to the extremely variable phenotype of *ADA2 *deficiency, the condition is underdiagnosed. In our case, the pathogenic variant in *ADA2 *possibly was responsible for autosomal recessive vasculitis, autoinflammation, immunodeficiency, and hematologic defects syndrome (VAIHS; OMIM: 615688). It is a complex systemic autoinflammatory disorder in which vasculitis, deregulated immune function, and/or hematologic abnormalities may predominate. Inflammatory features include intermittent fever, rash (often livedo racemose/reticularis), and musculoskeletal involvement (myalgia/arthralgia, arthritis, myositis). Hematologic disorders may begin early in life or in late adulthood and can include lymphopenia, neutropenia, pure red cell aplasia, thrombocytopenia, or pancytopenia. The clinical features are highly pleiotropic, and patients may present with only some of the main features.

One of the most prevalent clinical presentations of DADA2 is vasculopathy of small and medium arteries, affecting mainly the skin and central nervous system. The primary investigations indicated the inflammatory phenotype as the most prevalent. Yet, the present case did not have a comparable spectrum of disease manifestations, and he initially presented with immune thrombocytopenia and hemolytic anemia. In addition, 4% to 16% of the patients presented with hematological manifestations, combined with other symptoms, and the minority with severe anemia or pancytopenia as the primary or single signs [9] . The present case did not present with hypogammaglobulinemia or recurrent infections, although there was a decrease in IgA and IgM levels, supporting the assumption that *ADA2 *deficiency may cause a deficit in memory B cells.

Considering the homozygous likely pathogenic variant in *ADA2 *and the supportive phenotype of the patient, a genetic diagnosis of vasculitis, autoinflammation, immunodeficiency, and hematologic defects syndrome is very likely. To confirm the homozygosity of the detected variant, parental segregation analysis is recommended. Targeted molecular genetic testing and, if indicated, prenatal analysis can be offered to family members. We did not detect any pathogenic or likely pathogenic variants in the genes for which incidental findings are reported, based on the American College of Medical Genetics (ACMG) guidelines.

## Conclusions

This case was reported due to the finding of a new mutation that causes DADA2. Screening for CECR1 mutations should be included in the differential diagnosis of pediatric patients manifesting with chronic anemia, thrombocytopenia, or early-onset stroke, as the findings would have a direct impact on the clinical conclusion and outcome.
